# Smartphone App-Based Remote Monitoring Challenges in Patients with Cardiac Resynchronization Therapy Defibrillators—A Multicenter Study

**DOI:** 10.3390/jcm13216323

**Published:** 2024-10-23

**Authors:** Dagmar Kowal, Marek Prech, Agnieszka Katarzyńska-Szymańska, Artur Baszko, Grzegorz Skonieczny, Elżbieta Wabich, Maciej Kempa, Błażej Rubiś, Przemysław Mitkowski

**Affiliations:** 1 Department of Clinical Chemistry and Molecular Diagnostics, Poznan University of Medical Sciences, 60-806 Poznan, Poland; 85126@student.ump.edu.pl; 2 Doctoral School, Poznan University of Medical Sciences, 60-812 Poznan, Poland; 3 Department of Cardiology, Provincial Hospital, 64-100 Leszno, Poland; 4 1st Department of Cardiology, Poznan University of Medical Sciences, 60-355 Poznan, Poland; 5 2nd Department of Cardiology, Poznan University of Medical Sciences, 61-485 Poznan, Poland; 6 Department of Cardiology, Provincial Polyclinic Hospital, 87-100 Torun, Poland; 7 Department of Cardiology and Electrotherapy, Medical University of Gdansk, 80-210 Gdansk, Poland

**Keywords:** Bluetooth, cardiac resynchronization therapy, heart failure, remote monitoring, smartphone app

## Abstract

**Background/Objectives**: Remote monitoring (RM) cardiac implantable electronic devices for adults delivers improved patient outcomes. However, previously used bedside transmitters are not optimal due to deficient patient adherence. The goal of this study was to evaluate the efficacy of RM regarding the connectivity of smartphone app-based solutions, adherence to scheduled automatic follow-ups, and prevalence of alert-based events. **Methods**: We evaluated the adult heart failure (HF) population with an implanted cardiac resynchronization therapy defibrillator (CRT-D) divided into two arms: with app-based RM (abRM) and without app-based RM (control). **Results**: A total of 81 patients (median age of 69.0) were included in our study. Sixty-five patients received a CRT-D with abRM functionality, and sixteen did not. Twelve patients had no smartphone, and two provided no consent, resulting in their transfer to the control group. Finally, the abRM arm consisted of 51 patients, while 30 patients were in the control group. The median period of follow-up lasted 12 months. Among abRM patients, 98.0% successfully transmitted their first scheduled follow-up, and 80.4% were continuously monitored. Alert-based events were mainly related to arrhythmic events and device functionality with significantly shorter median times to notification (1 day vs. 101 days; *p* < 0.0001) in the abRM group. **Conclusions**: Our study showed a high level of compliance with timely initial transmission and adherence to scheduled remote follow-ups. Patient enrollment eligibility was a major challenge due to the limited accessibility of smartphones in the population. App-based RM demonstrated an accurate notification of events and patient-initiated transmissions in emergencies, regardless of location.

## 1. Introduction

Since 2015, remote monitoring (RM) has been recognized as a component of care for adult patients with implanted cardiac implantable electronic devices (CIEDs), and class I recommendation was granted by international physician societies [1,2]. The significant reduction in notification time from the onset of ventricular and supraventricular arrhythmias to their evaluation (including silent arrhythmias), the early detection of lead or device malfunction, and monitoring battery status were documented [3,4,5,6]. Commencing RM within 2–4 weeks of a new CIED implant and conducting remote, scheduled follow-ups every 3 months for adult patients is advised [2]. Daily remote checks of predefined alert events leading to prompt diagnoses of arrhythmic episodes or device-based events are essential to system functionality. Consequently, the organized and well-structured reaction of the dedicated medical team to the alerts recorded and available on the manufacturers’ servers results in earlier clinical responses, a reduction in inappropriate diagnoses, a reduction in the hospitalization rate, and the improvement of patient prognosis and clinical outcome, as emphasized in the latest consensus [2]. One of the critical factors in RM is compliance, defined as the total number of adult patients with CIEDs enrolled in RM, the execution of initial remote transmissions within 2–4 weeks of new CIED implantation, and adherence to automatic follow-up intervals according to guidelines. As emphasized, the final compliance and success rates of RM are affected by maintaining continuous connectivity between the CIED, transmitter, and manufacturer server [2]. Several studies revealed that RM based on bedside transmitters achieved suboptimal patient compliance, ranging from 53 to 79% [7,8]. The potential explanations for decreased adherence to the scheduled follow-up pattern were the transmitter type, patients’ socioeconomic status, age, gender, and geographic localization [9,10]. The implementation of smartphone app-based technology communicating with CIEDs via low-energy Bluetooth (BLE) protocols might offer solutions to the listed challenges. Consequently, applying the app-based solution presented a reduction in traditional bedside transmitters’ deficiencies, which resulted in improved patient engagement, compliance, and final rates of successfully delivered transmissions, ranging from 92% to 94.6% [11,12]. However, further evaluation is needed, especially examining the challenges faced by HF patients with an implanted cardiac resynchronization therapy defibrillator (CRT-D) using new technologies and the medical team conducting their supervision.

The goal of this study was to evaluate the efficacy of RM regarding the connectivity of smartphone app-based solutions, adherence to scheduled automatic follow-ups, and prevalence of alert-based events.

## 2. Materials and Methods

### 2.1. Patients

A prospective analysis was conducted on HF patients who had a CRT-D implanted between November 2020 and April 2024 at five hospitals: 1st and 2nd Department of Cardiology at Poznan University of Medical Science; Department of Cardiology and Electrotherapy at Medical University of Gdansk; Department of Cardiology at Provincial Policlinic Hospital in Torun; and Department of Cardiology at Provincial Hospital in Leszno. The indications for CRT were in accordance with the guidelines at the time of implantation [13]. Patient consent was collected. Patients were divided into two arms: implanted with a BLE-enabled CRT-D eligible for app-based monitoring (Neutrino NxT HF, Abbott, Plymouth, MN, USA) available in academic centers, and a control group without BLE capability in non-academic ones. Initially, patients were randomized based on clinical data. However, due to limited smartphone availability (which is underlined in Section 4), some patients had to be reclassified, resulting in a loss of comparability between groups. Of interest, despite the observed selection bias, patients’ characteristics were comparable to the population with CIEDs in real-world HF registries [3,5,7,14]. The goal was to accomplish a 2:1 propensity score match. During the study, only one manufacturer offered BLE-enabled devices on Polish market. RM was not reimbursed or academic centers had not yet signed a reimbursement agreement with the National Health Fund. Therefore, patients enrolled in RM received remote follow-up analysis as a part of a research project. Patients were enrolled in the RM system before discharge, at home, or during their first planned outpatient clinic visit. A combined follow-up protocol was initiated for RM patients: automatically, every 61 days, remote follow-ups with daily alert checks were carried out and regular, outpatient clinic visits took place every 6–12 months.

For control group patients, every 6–12 months, outpatient clinic follow-ups were conducted. During our study, no delegated medical team was established, as RM reimbursement was not yet introduced. The study’s primary investigator was supported in each hospital by a cardiologist, experienced in CRT-D implantation and follow-ups. Remote transmissions were regularly evaluated via the internet-accessible website (merlin.net, Abbott). If the loss of communication alert was delivered, a telephone call to the patient was made. During the enrollment procedure, a questionnaire was conducted to verify if the patient possessed a smartphone. Additionally, their level of familiarity with smartphones was measured by five questions, regarding app installation, text messaging, sending pictures, reading emails, and checking weather forecasts. No or one positive response was interpreted as low, 2–3 as moderate, and 4–5 as a high level of familiarity. The second questionnaire was used to evaluate patients’ experience in using the app and its four functions: manual transmission, device status, history of transmissions, and technical support. Both questionnaires were specifically designed for this study and were not pre-validated; hence, their design was simple and uncomplicated to make it easier for patients to choose. The questionnaires were reviewed by implanting physicians and were tested with the five pre-enrolment HF patients to confirm patients’ full understanding and ensure the device’s reliability and validity. The Supplementary Materials presents all questions from both surveys. Patients enrolled in RM received support for their CRT-D in terms of app pairing and operating the app, including instructions on manual transmission activation. The importance of daily connections conducted automatically at night was highlighted to patients in order to ensure the best adherence and compliance with the scheduled or alert transmissions. The analysis of parameters was performed as previously described [15]. Briefly, patient demographics, clinical data regarding cardiac diagnosis, indications for CRT-D implantation, RM compliance defined as the time to enrollment and the first transmission, and RM adherence defined as the number and regularity of planned follow-up transmissions were analyzed. Furthermore, the total duration of RM and the detection of alert-based and clinical events were investigated. The types of alert-based events were classified into groups with respect to device functioning (lead dysfunction, high pacing output, battery status) and arrhythmic events. Also, the time from RM enrollment to the first alert transmission was determined, and the time to notification, identified as the availability on the merlin.net platform, excluding weekends and holidays, was also reported. Additional outpatient or clinic appointments for a patient were organized if the justified alert-based transmission was triggered. In the control group, similarly, the time from implantation to the first event was measured, whereas the time to notification was calculated from the event occurrence to the patient appearance in the outpatient clinic for a scheduled visit or in the emergency facility if the event was symptomatic. The hospital records were the source of information regarding patient’s death.

All patients underwent thorough evaluation of RM efficacy to measure compliance, which was defined as the overall enrollment of CRT-D patients in RM, commencing RM transmission within 2–4 weeks of enrollment, and adherence to the planned, remote follow-up interval (61 days).

### 2.2. Statistics

Descriptive statistics were used to present data such as mean, standard deviation, or median with interquartile range (Q1, Q3), range, number of events, frequency of prevalence, and percentages. The normal distribution was verified by the Shapiro–Wilk test. The Mann–Whitney and Chi-square with Yates correction test were conducted to compare unpaired groups. All statistical analyses were performed in Tibco Statistica software version 13 (StatSoft, Tulsa, OK, USA). A two-tailed *p*-value < 0.05 was considered a statistically significant difference.

## 3. Results

**Figure 1:**
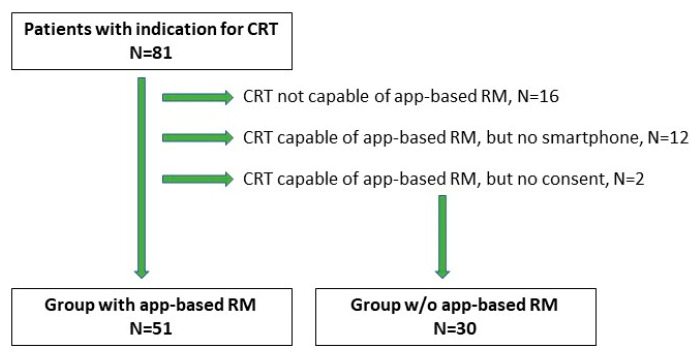
Cohort diagram of the study population.

A total of 81 patients were included in our study (74 males and 7 females). The median (Q1, Q3) patient age at implantation was 69.0 (60, 74) years. Baseline patients’ characteristics are available in Table 1. The group of 65 patients (80.2%) received a CRT-D with app-based RM functionality, and 16 patients (19.8%) without. In the first group, twelve patients had no smartphone and two patients did not provide consent, resulting in their transfer to the control group. Thus, the RM arm consisted of 51 patients, while 30 patients were in the control group (Figure 1). Among 51 patients in the RM arm, 11 had decided to purchase a smartphone to be eligible for active monitoring.

Patients were followed up remotely over a median period of 12 (5, 24) months. The control group was observed in the outpatient clinic over a median period of 27 (24, 30) months. During the follow-up, no patient deaths were recorded in the RM group and two patient deaths in the mechanism of HF progression in the control group. Control group patients had lower LVEF, a higher incidence of ischemic etiology, and a longer median follow-up time. No statistical significance was shown (*p* = 0.260) that could be related to the limited sample size or follow-up time. No deaths were related to device dysfunction (Table 2).

### 3.1. Compliance with App-Based RM

The median (Q1, Q3) time from CRT-D implantation to remote monitoring enrollment was 1 day (1, 7). There were 36 patients (70.6%) with successfully paired apps and who had initiated monitoring before discharge. The remaining patients (29.4%) were enrolled in RM via a telephone call, with family assistance while staying at home, except one patient who was paired at an outpatient clinic. The main reasons for the delay were the lack of adequate smartphones followed by an intentional purchase, insufficient Internet coverage at the hospital, or deficient rights of the user’s phone.

**Figure 2:**
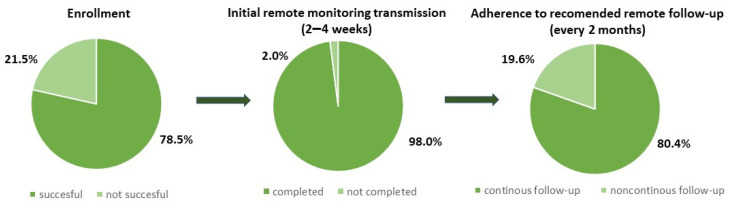
App-based remote monitoring compliance in adult patients with cardiac resynchronization therapy defibrillators.

Out of 65 patients who received a CRT-D capable of app-based RM, 51 patients (78.5%) were successfully enrolled in the RM system. Twelve patients who had no smartphone nor any intention of upgrading and two others who did not provide consent were transferred to the control group. The most common causes of RM ineligibility were socioeconomic status and concomitant, age-related diseases such as dementia, stroke, and eye diseases. In the RM arm, 50 patients (98.0%) had their first scheduled follow-up successfully sent. The group of 41 patients (80.4%) were continuously monitored during the observation, and all follow-up transmissions were delivered according to schedule (Figure 2).

**Figure 3:**
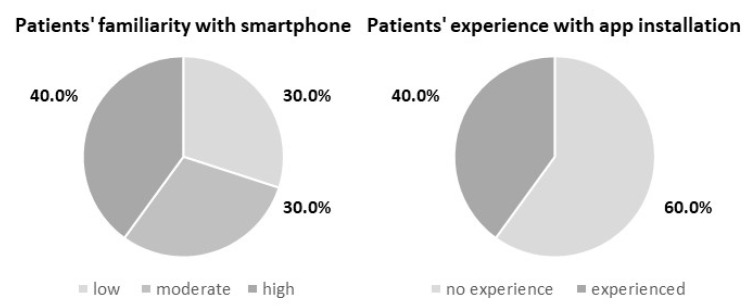
Familiarity and experience of patients with regard to smartphone functionality in the group of patients with noncontinuous remote follow-up. The mean age of patients with low smartphone familiarity was 81.0 years.

Further analysis of the recruitment questionnaire demonstrated that all patients who purchased the smartphone fulfilled the criteria of being continuously monitored, whereas among the group of 19.6% of patients with missed scheduled transmissions, three patients possessed low familiarity, three possessed moderate, and four possessed a high level of familiarity with smartphones. In this group, 6 out of 10 patients expressed no experience in app installation (Figure 3). Patients with mean age of 81.0 presented low familiarity with required technology. The evaluation of patients’ app experience questionnaire revealed that 84.0% of patients who had activated the app and had their first scheduled follow-up successfully sent used mainly two out of the four app functionalities: transmissions history and device status. Our data did not report an excessive use of the manual transmission feature. Out of twenty-two patients who sent intentional transmissions, four executed it more than five times, one was prompted by a medical team, and seventeen occurred during the first month of enrollment.

Our analysis also exposed accidental BLE disconnection or turning off the RM app as the most common cause of decreased connectivity. A group of RM patients contacted us directly and asked for assistance in repairing the app while changing or upgrading their phone, but events were solved ad hoc and not recorded; therefore, the data are incomplete.

### 3.2. RM Alert-Based Transmissions vs. Control Group

In the app-based RM group, 42 events were observed in 25 patients (49.0%) as follows: 88.1% were appropriately diagnosed, followed by a justified alert transmission, and 11.9% showed a false positive notification. The median (Q1, Q3) time to the first transmission was 158 (19, 372) days, and the median time to notification was 1 (1, 2) day. Further analysis of the appropriately detected alert-based events showed that 20 were related to arrhythmic events and 22 to device functioning (Table 3).

**Figure 4:**
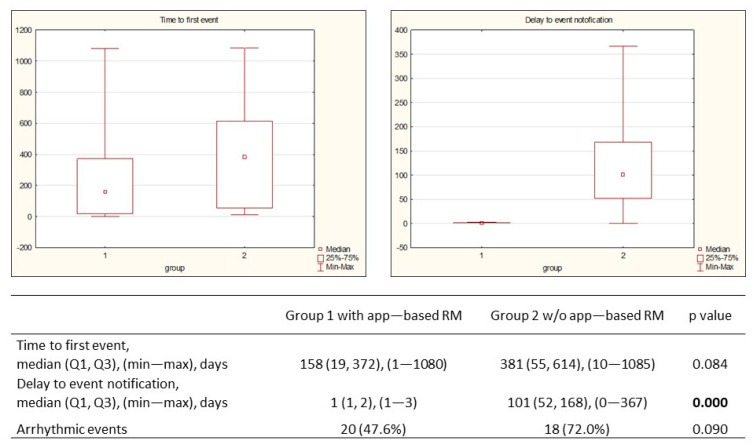
Time to first event and time to notification.

Similar analyses were conducted for the control group with respect to arrhythmic and device functioning events (Table 4). The median time to the first event was 381 (55, 614) days, and the median notification delay was 101 (52, 168) days. Although there was a difference in the occurrence of first events in both groups, no statistical significance was found (*p* = 0.084). A shorter median period to notification in the app-based RM group was noted: 1 day vs. 101 days (*p* = 0.0001) (Figure 4).

## 4. Discussion

In this first Polish real-world, prospective, multicenter study on the efficacy of smartphone app-based RM in HF patients with CRT-Ds, we showed a high level of connectivity as evidenced by good compliance with timely initial transmissions (98.0%) and adherence to scheduled remote follow-ups (80.4%). According to the guidelines, the RM should be regarded as a standard of care in HF patients with CIEDs. The patient enrollment and commencing RM transmission should be conducted within 2–4 weeks of implantation or device exchange, followed by scheduled remote follow-ups every 3 months and every 12 months for outpatient clinic visits [2]. Altogether, in our study, we met the requirements of the swift activation of RM, but the patient’s enrollment outcome was suboptimal. The 78.5% success rate was mainly influenced by socioeconomic status and concomitant diseases, which resulted in a lack of smartphone to download the monitoring app and a lack of an understanding of the clinical benefits of RM. Similar conclusions were recently presented from 10 Italian hospitals and 495 patients (67 ± 13 years), where 84% had access to smartphones, 85% were willing to use the HF monitoring app, but only 63% downloaded the app [16]. This shows that the challenges we encountered during patient enrollment to RM are comparable in the European Union region. Therefore, we should consider optimized patient selection for remote monitoring programs to achieve a high level of connectivity, utilizing the latest communication technologies coupled with growing patient awareness and confidence in using informative communication platforms, as was recently emphasized by Tedeschi A et al. [17].

An important role in the high enrollment ratio is not only the medical team but also family assistance with the patient’s app in the CIED pairing process. In our group, 29.4% of patients executed pairing at home with family support. This shows that this process could be performed out of the clinic, possibly with phone support. During the study, we noticed the growing patient awareness of RM advantages, mainly those related to reimbursement introduced in mid-2023 and broadly covered by media. This resulted in 11 patients purchasing a smartphone to use beneficial app-based RM. At the same time, technological headway makes smartphones more common among seniors, and current smartphone users will become older; therefore, the challenges with suboptimal eligibility will gradually decline. The pace of technology adoption can be geography- and socioeconomics-related. The recent study conducted on a group exceeding 18,000 US patients demonstrated an enrollment level of 94.4% in the app-based group and 85.0% in the bedside transmitters group [18]. The remaining group of technologically excluded patients should be offered different solutions for RM, such as mobile transmitters or traditional bedside transmitters, as provided by manufacturers. However, the disadvantages of immobile solutions should be taken into consideration. In our previous study with pediatric patients with implanted pacemakers and bedside transmitters, the enrollment success rate was 100.0%, as each patient received a paired transmitter at discharge or in an outpatient clinic. Unfortunately, the adherence was suboptimal, especially during the holiday season, when 34.8% of patients missed scheduled transmissions [15].

In our study, 98.0% of patients had the first planned follow-up successfully sent, and 80.4% of patients participated in continuous surveillance without losing scheduled transmissions, which were planned every 61 days. This reveals that smart and mobile technologies solve connectivity challenges in RM, primarily related to bedside systems. These results are in concordance with recently published data presenting adherence in app-based solutions in the range of 92.0–94.6% [11,12,18].

Acknowledging the patient experience of using an app is crucial for final compliance. We noticed increased patient engagement in the therapy process via app-built functionality. Patients’ experience evaluations revealed that scheduled transmission history and battery status were the most frequently and intentionally used app features. Furthermore, no excessive use of the manual transmission feature creating a work overload for the medical team was observed among monitored patients. Patients also contacted research team members for support in maintaining and continuing RM when their smartphone was upgraded and the repairing security code was lost. This demonstrates patients’ perceptions of safety and their understanding of continuous RM for long-term clinical outcomes. Similar insights into the patient experience emerge from recent studies [12,18,19,20]. Furthermore, the patients’ perception of remote and virtual visits has changed since COVID-19. The Italian researchers reported not only increased patient satisfaction from virtual visits, but also a preference for virtual visits over in-clinic ones among 86.4% of examined patients. Importantly, the remote management strategy presented in that study reported no significant differences in clinical outcomes in comparison to in-clinic visits [21].

### Alert-Based Transmissions

Remote monitoring is recommended for the early detection of arrhythmic events, especially asymptomatic ones, and device and lead dysfunction [3]. Our evaluation presented that 88.1% of events in the app-based RM group were appropriately diagnosed with a median time of 158 (19, 372) days. The median time to events in the control group was twice as long (158 vs. 381). In our opinion, the presented difference (statistically insignificant) may result from a limited number of the study group and a mismatch in the percentage of ischemic and non-ischemic cardiomyopathies of baseline patients characteristics. However, this ischemic substrate variance does not influence the time from event occurrence to notifying the medical team, which is directly related to the patient monitoring method. Our findings revealed a median notification delay of 1 (1, 2) day in the app-based RM group and 101 (52, 168) days in the control group. The prompt notification reported in the app-based RM group can potentially reduce the risk of adverse events from silent arrhythmias, reduce hospitalization time, reduce HF decompensation, and prevent its recurrence. The observed proportions of notification time in the studied population are in accordance with the results of the previously published Connect Trial (4.6 vs. 22 days) and the Evolvo Study (1.4 vs. 24.8 days), respectively [5,22]. As the time to notification in our study in the RM arm is improved, it implicates that app-based technology may offer superior efficacy to bedside transmitters in the rapid detection of alert-based events. Other reports show that app-based technology provides better connectivity than bedside transmitters [11,12,18] mainly due to their lower mobility, which may pose a risk of decreased success rates.

However, with respect to delayed notification times, the meta-analysis conducted by Parthiban et al. presented a significant mean difference in the days to clinical decision/event detection of 27.1 days [23]. The difference presented in our research is larger and may result from the limited study population or the lack of sufficient data regarding medical decisions from the implanting center. Nevertheless, the main study goal was to evaluate the efficacy of the app-based RM solution as an alternative to the traditional bedside system, not to conventional in-office follow-ups. The superiority of RM has been well analyzed and is reflected in the 2023 HRS/EHRA/APHRS/LAHRS expert consensus [2].

The improved patient engagement due to available smartphone-based technologies and enhanced communication capabilities between the patient and the medical team were presented recently in a case report. Both the swift diagnosis of silent AF and the confirmation of terminated VF via manual transmission followed by hospital admission were described [24].

## 5. Limitations

The technology examined in our study was commercially introduced in the European Union in November 2020. Throughout the entire period of our research, only one manufacturer commercially offered BLE-enabled CRT-Ds in Poland; therefore, the analysis could not be extended to other devices with respect to the RM arm. Furthermore, RM was not reimbursed for most of our study period. Therefore, BLE-enabled CRT-Ds were implanted only in academic centers, and patients enrolled in RM received remote follow-up analysis as a part of a research project, but not from a dedicated hospital unit. Patients who did not qualify for the RM arm (no smartphone or no consent) were moved to the control group at the discretion of the implanting physician. Therefore, we cannot completely rule out selection bias. The process of patients’ reclassification resulted in the loss of balance between the groups with respect to clinical data and HF etiology. It seems unlikely, however, that the observed disproportion in clinical data may influence both the measured reaction time to alert events and the evaluation of app-based RM efficacy. Although the number of participants was calculated at the beginning of the study, the patients’ enrollment was influenced by the COVID-19 pandemic and impacted sample size.

## 6. Conclusions

Our real-world, prospective, multicenter study showed a high level of connectivity enabled by the good compliance with timely initial transmissions (98.0%) and adherence to scheduled remote follow-ups (80.4%). Patient enrollment eligibility was a major challenge due to the limited accessibility of smartphones in the population, but it will likely improve as the technology becomes more widespread. App-based RM demonstrated the ability to provide swift and accurate notifications of alert-triggered events and patient-initiated transmissions in emergencies, regardless of location.

Our findings are consistent with previously published results regarding CIEDs and provide broader insights into the situation of heart failure patients monitored with smartphone apps, identifying key areas for improvement.

## Figures and Tables

**Table 1:** Baseline characteristics of patients.

	Study Cohort	Group with App-Based RM	Group *w*/*o* App-Based RM	*p* Value
	N = 81	N = 51	N = 30	
Age, median (Q1, Q3), years	69.0 (60, 74)	67.5 (57, 75)	69.0 (64, 72)	0.924
Male gender	73 (90.1%)	46 (90.2%)	27 (90.0%)	0.721
Cardiomyopathy:				
ischemic	46 (56.8%)	24 (47.1%)	21 (70.0%)	0.076
nonischemic	31 (38.3%)	25 (49.0%)	7 (23.3%)	0.041
other	4 (4.9%)	2 (3.9%)	2 (6.7%)	0.984
NYHA class II or III	64 (79.0%)	46 (90.2%)	19 (63.3%)	0.008
History of atrial fibrillation/atrial flutter	17 (21.0%)	13 (25.5%)	4 (13.3%)	0.310
ICD indication: primary prevention	64 (79.0%)	41 (80.4%)	23 (76.7%)	0.908
LV ejection fraction (%)	30.0 (25–35)	30.0 (25–35)	25.0 (20–30)	0.026
LBBB	71 (87.7%)	46 (90.2%)	25(83.3%)	0.578
Diabetes mellitus	26 (32.1%)	13 (25.5%)	13 (43.3%)	0.157
Chronic renal failure	28 (34.6%)	17 (33.3%)	11 (36.7%)	0.950
CABG	7 (8.6%)	4 (7.8%)	3 (10.0%)	0.940
NT-proBNP, median (Q1, Q3), (pg/mL)	1818.0 (543, 3362)	1291.0 (449, 2985)	2500.5 (1349, 5456)	0.111
CRP, median (Q1, Q3), (mg/L)	4.0 (2, 8)	4.0 (2, 8)	4.0 (2, 8)	0.993

Legend: NYHA—New York Heart Association, ICD—implantable cardioverter-defibrillator, LV—left ventricle, LBBB—left bundle brunch block, CABG—coronary artery bypass graft.

**Table 2:** Remote monitoring and control group characteristics.

Number of patients capable of app-based RM	65
Number of patients enrolled in RM	51 (78.5%)
Patients:	
no smartphone	12 (18.5%)
no consent	2 (3.1%)
purchased smartphone	11 (16.9%)
RM follow-up time, median (Q1, Q3), (min–max), months	12 (5, 24), (1–40)
Patients with at least 1 FU after RM enrollment	50 (98.0%)
Continuous adherence to RM FU scheme	41 (80.4%)
Actively monitored patients at final analysis	38 (74.5%)
Control group follow-up time, median (Q1, Q3), (min–max), months	27 (24, 30), (11–41)
Patients died in monitored group	0
Patients died in control group	2

Legend: RM—remote monitoring, FU—follow up.

**Table 3:** Remote monitoring alert-based transmissions.

Number of Patients with RM	51	Diagnosis	CRT-D Diagnostics	Corrective Action
Patients with first alert transmission	25 (49.0%)			
First alert transmissions	42			
Alert types:				
VF	3 (7.1%)	Ventricular fibrillation	IEGM	1 VF—hospitalization, 2 VFs—inappropriate detection of AF with fast V conduction; device reprogramming and medication adjustment
VT	1 (2.4%)	Ventricular tachycardia	IEGM	Medication adjustment
nsVT	10 (23.9%)	Ventricular non-sustained arrhythmias	HVR histogram + IEGM	Medication adjustment
AMS	6 (14.3%)	Atrial fibrillation/atrial flutter	AMS histogram + IEGM	4 AF episodes, 2 AFl episodes, medication adjustment, and/or cardioversion
BiV less then limit	2 (4.8%)	Premature ventricular complexes	Rhythm diagnostics + counters + histogram	Medication adjustment
High capture output	3 (7.1%)	RV/LV pacing deficit	Real time IEGM + high capture output + threshold trend	Device reprogramming, observation
Sense amplitude below threshold	3 (7.1%)	A/V signal drop	Automatic signal measurements + trend	2 in A channel and 1 in V channel, observation
Non-sustained V oversensing	11 (26.2%)	Intermittent post BiV T-wave oversensing	IEGM	Device reprogramming
AMS due to oversensing	3 (7.1%)	Farfield, V oversensing in atrial channel	AMS IEGM + oversensing evaluation	Device reprogramming

Legend: RM—remote monitoring, CRT-D—cardiac resynchronization therapy defibrillator, VF—ventricular fibrillation, VT—ventricular tachyarrhythmia, nsVT—none sustained ventricular tachyarrhythmia, AF—atrial fibrillation, AFl—atrial flutter, IEGM—intracardiac electrogram, HVR—high ventricular rate, AMS—auto mode switch, BiV—biventricular, A—atrial, V—ventricular, RV—right ventricle, LV—left ventricle.

**Table 4:** Control group event episodes.

Number of Patients *w*/*o* RM	30	Diagnosis	CRT-D Diagnostics	Corrective Action
Patients with first event episodes	20 (66.7%)			
First event episodes occurrence	25			
Events types:				
VT	3 (12.0%)	Ventricular tachycardia	IEGM	Medication adjustment
nsVT	8 (32.0%)	Ventricular non-sustained arrhythmias	HVR histogram + IEGM	Medication adjustment
AMS	8 (32.0%)	Atrial fibrillation/atrial flutter/atrial tachycardia/oversensing	AMS histogram + IEGM	1 AF episode, 1 AFl episodes, 5 AT episodes, medication adjustment and/or cardioversion, observation, 1 Far R oversensing resulted in device reprogramming
Sense amplitude below threshold	2 (8.0%)	V signal drop	automatic signal measurements + trend	2 in V channel, observation
Non-sustained V oversensing	2 (8.0%)	Intermittent post BiV T-wave oversensing	IEGM	Device reprogramming
Lead impedance, noise	2 (8.0%)	RV lead disfunction	Impedance trend, IEGM noise	1 RV lead replacement, 1 observation

Legend: RM—remote monitoring, CRT-D—cardiac resynchronization therapy defibrillator, VT—ventricular tachyarrhythmia, nsVT—none sustained ventricular tachyarrhythmia, AF—atrial fibrillation, AFl—atrial flutter, IEGM—intracardiac electrogram, HVR—high ventricular rate, AMS—auto mode switch, BiV—biventricular, V—ventricular, RV—right ventricle.

## Data Availability

Please use sentence: Data are contained within the article and Supplementary Materials.

## Supplementary Materials

The following supporting information can be downloaded at https://www.mdpi.com/article/10.3390/jcm13216323/s1, Patient questionnaire—2020–2024.
