# Supplementary material for: Smartphone App-Based Remote Monitoring Challenges in Patients with Cardiac Resynchronization Therapy Defibrillators—A Multicenter Study
Source: J Clin Med. 2024 Oct 23;13(21):6323. doi: 10.3390/jcm13216323 (PMC11545944; doi:10.3390/jcm13216323)
Supplement: Supplementary file 1 [file jcm-13-06323-s001.zip › jcm-3258678-supplementary.pdf]

Patient questionnaire – 2020-2024

Part 1 - after implant, before discharge

1.	Do you have a smartphone?	yes	no
	If "YES" move to next questions		
2.	Do you install additional applications on your smartphone?	yes	no
3.	Do you send text messages?	yes	no
4.	Do you send photos?	yes	no
5.	Do you send and read emails?	yes	no
6.	Do you check the weather forecast?	yes	no

Part 2 – patients' experience in using the MyMerlinPulse app (only for RM group)

1. Is using the telemonitoring application difficult? 1 2 3 4 5

   (1 – not at all, 2 – a bit, 3 – medium difficulty, 4 - difficult, 5 – very difficult)

2. Which application features did you use?

   - Manual transmission, also called as patient initiated transmission

   - Device status

   - History of transmissions

   - Technical support
